# Comparison of Marginal Fracture Between Conventional and Bonded Amalgam Restorations in Posterior Permanent Molar Teeth

**DOI:** 10.7759/cureus.44295

**Published:** 2023-08-28

**Authors:** Humara Iqbal, Sadiq Amin Ahmed Rana, Afshan Manzoor, Amara Nazir, Mobeen Akhtar, Hareem Ghaffar, Muhammad Kashif

**Affiliations:** 1 Operative Dentistry, Bakhtawar Amin Medical and Dental College, Multan, PAK; 2 Operative Dentistry, Nishtar Institute of Dentistry (NID), Multan, PAK; 3 School of Dental Sciences, Universiti Sains Malaysia, Kota Bharu, MYS; 4 Oral Pathology, Bakhtawar Amin Medical and Dental College, Multan, PAK

**Keywords:** dental resin, bonded amalgam, direct restoration, dental amalgam, carious permanent molars

## Abstract

Background/objectives

Dental amalgam has been a successful restoration for over a century. However, restoration failures due to secondary caries, fractured teeth or restorations, marginal deficiencies, tooth wear, and secondary caries remain significant concerns. Amalgam-bond, known for its ability to bond amalgam to the tooth structure and prevent percolation, forms a strong bond with vital dentin. This study aimed to compare the outcome of marginal fractures in bonded amalgam and conventional amalgam posterior restorations among patients at a tertiary care dental hospital.

Materials and methods

Sixty consecutive patients aged 25-35 years, meeting the inclusion and exclusion criteria, participated in this study. A thorough history, clinical examination, and standardized periapical radiographs were conducted. Patients were divided randomly into two equal groups, group A and group B. Group A received bonded amalgam restorations, while group B received conventional amalgam restorations. Polishing was performed at a recall visit after seven days, and a follow-up evaluation was done after two months. The final assessment of marginal fractures was recorded after six months.

Results

After six months, 28 (46.7%) patients showed no marginal fractures, including 11 males and 17 females. On the other hand, 32 (53.3%) patients exhibited marginal fractures, comprising 17 males and 15 females. The clinical success rate of group A was better than group B (*p* = 0.001).

Conclusion

Bonded amalgam demonstrates a high success rate and should be a routine choice for treating carious permanent molars in dental practice.

## Introduction

Dental amalgam, the most commonly used restorative material, has a history of more than 150 years. It is a combination of a silver alloy and mercury. The silver alloy, mainly comprising silver, tin, and copper and sometimes including zinc, palladium, or indium, undergoes a chemical reaction with mercury to form dental amalgam. This reaction is known as the amalgamation reaction. Despite the hazardous nature of certain mercury forms, the mercury in amalgam is securely bound to other metals, ensuring its stability and safety for dental applications [1,2]. Dental amalgam is a highly versatile restorative material widely employed in dentistry, making up about 75% of all restorative materials used by dentists. Currently, there are no satisfactory economical substitutes for dental amalgam, mainly due to its unmatched blend of dependable long-term performance in load-bearing scenarios and cost-effectiveness. Its applications are diverse, benefiting from attributes like low technique sensitivity, self-sealing properties, and extended durability [3,4].

While there is evidence of a decline in global usage, the cost-effectiveness, durability, and ease of manipulation of dental amalgam continue to persuade many dentists to prefer it as their primary choice for restoring posterior teeth. However, careful consideration is essential in diagnosing the appropriate type of restoration required. In cases where significant loss of tooth structure requires support from the restoration, a gold inlay might be recommended, although extensive amalgam restorations can also be a viable option. Dental amalgam finds application on all surfaces of posterior teeth and occasionally in the lingual pits of anterior teeth, constituting a significant portion of all dental restorations. Despite its popularity, the silvery-gray appearance of amalgam limits its use to posterior teeth. Surveys have indicated a lower percentage of failures among amalgam restorations compared to other restorative materials. Nevertheless, when failures do occur, they are often attributed to improper cavity preparation design and faulty material manipulation. Therefore, every step, from selecting the alloy to polishing the restoration, significantly impacts the physical properties and potential success or failure of the restoration [1,3,5].

Conventional amalgam serves as a filling material that merely occupies the prepared cavity space, failing to restore the tooth's fracture resistance lost during cavity preparations. Moreover, ensuring sufficient resistance and retention form for amalgams might involve removing healthy tooth structure. Additionally, due to the lack of bonding between amalgam and tooth structure, microleakage is inevitable immediately after placement. To address these drawbacks, adhesive systems that reliably bond to enamel and dentin have been introduced. One such system is the amalgam bond, which relies on a dentinal bonding approach developed by Nakabayashi et al. in Japan. Studies have recorded varying bond strengths, typically around 12-15 MPa, which appear to be consistently achievable. In a study using bonded amalgam with spherical amalgam, Shen et al. reported a mean bond strength of 27 MPa [6,7].

Adhesive techniques are now widely employed in various restorative materials, including amalgam. Multiple generations of dentine bonding agents have been developed, primarily for bonding composite resins. When adhesive bonding is utilized for amalgam restorations, it reduces the requirements for retention and resistance, leading to improved sealing. To cater to amalgam bonding requirements, specific products have been developed. Recently, some dental adhesive resins have exhibited remarkable adhesive properties, effectively bonding to both teeth and amalgam [7-9].

Amalgambond, developed in Japan by Nakabayashi et al. over a decade ago, is based on a dentinal bonding system. It involves using a 10% citric acid and 3% ferric chloride solution to remove the smear layer and demineralize the dentine surface. Subsequently, a primer is applied to the conditioned dentine, and a self-curing methacrylate resin containing the adhesive monomer 4-META is used to impregnate the primed dentine. In an in vitro study, the q1000 effect of bonding amalgam restorations using the bonding agent Syntac and the adhesive resin luting agent Resinomer was evaluated in maxillary premolars with mesio-occluso-distal preparations. The results revealed that using resinomer as an amalgam bonding agent in mesio-occlusal-distal (MOD) amalgam restorations increased the fracture resistance of the tooth [10,11]. The aim of the present study is to compare the marginal fracture between conventional and bonded amalgam restorations in posterior teeth.

## Materials and methods

Study design and setting

This interventional study (prospective) was conducted at the Department of Operative Dentistry, Bakhtawar Amin Dental College and Hospital (BADC&H), Multan, following approval (REU/DSG/09/706/) from the Institutional Research Board (IRB) of BADC&H, Multan, Pakistan.

Sample size and selection

Sixty participants were included in the study, with 30 individuals in each group (groups A and B). Sample selection was carried out using a non-probability purposive sampling technique. Eligible participants of both genders, aged between 20 and 35 years, with class I carious lesions on the occlusal surfaces of either maxillary or mandibular molars were recruited. Patients with class I cavities that were too deep with thin margins, mobile teeth, difficult isolation, malocclusion, or co-morbid conditions like uncontrolled diabetes or unstable angina were excluded from the study.

Data collection procedure

Patients were recruited from the Outpatient Department of Operative Dentistry, BADC&H, Multan, after obtaining informed consent and taking a detailed medical history. All patients were selected based on the predefined inclusion and exclusion criteria. Random allocation to either group A or B was performed using a lottery method. In group A, cavities were filled with bonded amalgam, while group B received conventional amalgam restorations. The cavity preparation involved using a medium-grit diamond cylindrical fissure bur, followed by a fine-grit bur for finishing. In cases of bonded amalgam restoration, a bonding agent (Adper ScotchBond Multi-Purpose Dual Cure, 3M ESPE, USA) was applied according to the manufacturer's instructions.

**Figure 1:**
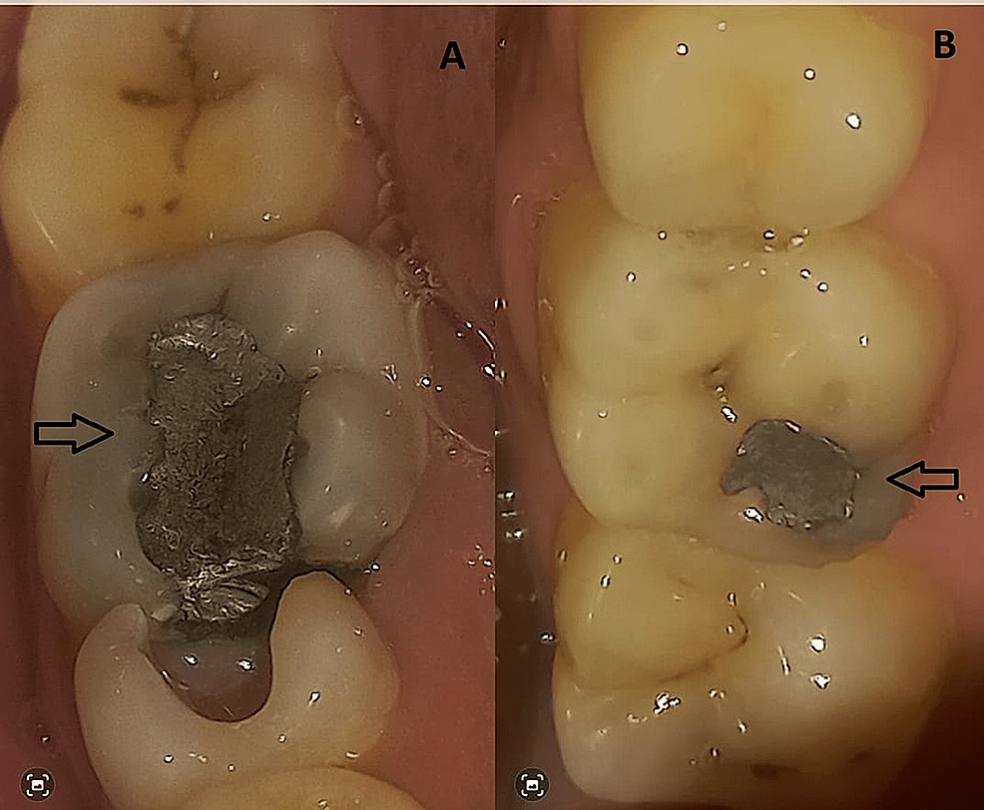
(A) The class 2 conventional amalgam filling, showing signs of marginal fracture, wear and tear, has developed a ditch in its occlusal (biting) area. (B) The class 1 conventional amalgam filling showing poor marginal adaptation, recurrent caries, marginal fracture and amalgam blues.

Mechanically triturated amalgam was condensed and adapted to the cavity margins for bonded amalgam restorations. For conventional amalgam restorations, the lining was applied when necessary after cavity preparation. All procedures were performed by researchers under the supervision of an experienced consultant restorative dentist to minimize bias. Patients were given appropriate instructions, and restorations were polished the next day using a standard polishing system. Follow-up visits were scheduled after seven days and at two-month intervals. The final outcome, i.e., the presence or absence of a marginal fracture, was evaluated after six months (Figure 1). Marginal fractures were assessed and recorded using modified United States Public Health Service (USPHS) criteria as follows. Marginal fracture: (i) alpha - no defect; (ii) beta - minor defect; (iii) Charlie - major defect; (iv) delta - margin fracture.

Data analysis

Data from both groups were entered into SPSS version 25.0 for analysis (IBM Corp., Armonk, NY). Frequencies and proportions were presented for qualitative variables such as gender, type of restoration used, and location of the tooth. The mean and standard deviation were presented for quantitative variables, such as age. The Chi-square test was applied, and marginal fracture (yes/no) was compared between both groups. A p-value of ≤0.05 was considered statistically significant. Confounding variables, such as age and gender, were controlled by stratification during the analysis.

## Results

A total of 60 patients participated in this study, with each group consisting of 30 patients. In group A, the mean age of the patients was 30.36 ± 3.44 years, ranging from 25 to 35 years. For group B, the mean age was 30.33 ± 2.64 years. The minimum age observed in group B was 26 years, and the maximum age was 35 years, as depicted in Table 1.

**Table 1:** Descriptive statistics of age

Restorative material				Statistic
Age	Group A	Mean		30.3667
		95% Confidence interval for mean	Lower bound	29.0788
			Upper bound	31.6545
		5% trimmed mean		30.4074
		Median		30.5000
		Variance		11.895
		Std. deviation		3.44897
		Minimum		25.00
		Maximum		35.00
	Group B	Mean		30.3333
		95% confidence interval for mean	Lower bound	29.3462
			Upper bound	31.3205
		5% trimmed mean		30.3148
		Median		30.0000
		Variance		6.989
		Std. deviation		2.64358
		Minimum		26.00
		Maximum		35.00

Out of the total 60 participants, 28 (46.66%) were males and 32 (53.33%) were females. In group A, there were 12 males (42.9%), while group B had 16 males (57.1%). Similarly, there were 18 females in group A and 14 females in group B, as shown in Table 2.

**Table 2:** Distribution of gender in both groups N: frequency, %: percentage

Gender	Restorative material		Total N (%)
	Group A N (%)	Group B N (%)	
Male	12 (42.9%)	16 (57.1%)	28 (100.0%)
Female	18 (56.3%)	14 (43.8%)	32 (100.0%)
Total	30 (50.0%)	30 (50.0%)	60 (100.0%)

After a six-month follow-up period, 28 patients (46.7%) exhibited no fractures. Among them, 21 (70.0%) belonged to group A, and 7 (23.3%) were from group B. In contrast, 32 patients (53.3%) experienced marginal fractures after six months, with 9 (30%) in group A and 23 (76.7%) in group B (Figure 1).

The Chi-square test revealed a highly significant value of 0.001, indicating the difference between the two groups, as outlined in Table 3.

**Table 3:** Distribution of marginal fracture after six months between groups p ≤ 0.05 significant, N: frequency, %: percentage

Restorative material groups	Marginal fracture after 6 months		Total N (%)	p-value
	No fracture N (%)	Yes fracture N (%)		
Group A	21 (70.0%)	9 (30.0%)	30 (100.0%)	0.001
Group B	7 (23.3%)	23 (76.7%)	30 (100.0%)	
Total	28 (46.7%)	32 (53.3%)	60 (100.0%)	

Upon stratifying the occurrence of marginal fractures by age, two age groups emerged: 20-27 years and 28-35 years. In the 20-27-year-old age group, 5 patients exhibited marginal fractures, while in the 28-35-year-old age group, 27 patients experienced such fractures. This discrepancy might be attributed to changes in dietary patterns with increasing age. Among the 28 patients without marginal fractures, 7 were in the 20-27 year age group, and 21 were in the 28-35 year age group, as indicated in Tables 4-5. 

**Table 4:** Distribution of marginal fracture after six months between both genders p ≤ 0.05 significant, N: frequency, %: percentage

Gender	Marginal fracture after 6 months		Total N (%)	p-value
	No fracture N (%)	Yes fracture N (%)		
Male	11 (39.3%)	17 (60.7%)	28 (100.0%)	0.001
Female	17 (53.1%)	15 (46.9%)	32 (100.0%)	
Total	28 (46.7%)	32 (53.3%)	60 (100.0%)	

**Table 5:** Stratification of marginal fractures after six months in both age groups p ≤ 0.05 significant, N: frequency, %: percentage

Age group	Marginal fracture after 6 months		Total	p-value
	No fracture N (%)	Yes fracture N (%)		
20–27 years	7 (58.3%)	5 (41.7%)	12 (100.0%)	0.005
28–35 years	21 (43.8%)	27 (56.3%)	48 (100.0%)	
Total	28 (46.7%)	32 (53.3%)	60 (100.0%)	

## Discussion

The long-term success of extensive amalgam restorations depends on effectively preventing the major traditional mechanical issues associated with them, such as marginal fracture, bulk fracture, and tooth fracture. The survival and durability of these restorations are greatly influenced by the composition of the alloy, particularly the levels of zinc and copper. The corrosion resistance of the amalgam is affected by these elements, and higher copper content in high-copper amalgams leads to better survival rates compared to conventional amalgams [12,13].

Letzel conducted a retrospective study to examine the survival and failure patterns of amalgam restorations. The primary modes of failure observed were bulk fracture (4.6%), tooth fracture (1.9%), and marginal ridge fracture (1.3%). Additionally, 0.8% of the restorations failed due to various other reasons [13,14].

This study aimed to compare the retentive strength and durability of bonded amalgam restorations with those of non-bonded ones. The in vivo research evaluated the effectiveness of an adhesive resin (ScotchBond Multi-Purpose, dual cure) as a bonding agent for amalgam restorations. Prior to this study, the routine clinical use of this adhesive resin for bonding amalgam restorations had been suggested [14-16].

The findings revealed a statistically significant distinction between the two groups: bonded amalgam restorations and conventional restorations at the six-month mark. In group A (bonded amalgam group), 17% of cases exhibited continuous amalgam with adjacent tooth structure when a sharp probe was moved along the margins. In 22% of cases, no visible evidence of crevice formation was observed, but the probe could penetrate. Additionally, 8% of cases showed visible evidence of a crevice that the probe penetrated, while 3% displayed visible evidence of a crevice with exposure of underlying dentine or base.

On the other hand, in group B (the conventional amalgam group), none of the cases demonstrated continuous amalgam with adjacent tooth structure when using a sharp probe along the margins. In 18% of cases, no visible evidence of gap formation was noticed, but the probe was able to penetrate. Moreover, 20% of cases showed visible evidence of a crevice into which the probe could penetrate, and in 12% of cases, a visible crevice was present with exposure to underlying dentine. Group A outperformed group B overall. After six months, marginal fractures were less frequently observed, with only 11% occurring in group A compared to 31% in group B.

The conventional amalgam restorations without any bonding agent exhibited significantly lower retention values compared to the bonded groups. This finding aligns with the results obtained by Dewaele et al., despite differences in bonding materials and experimental methods. Bonded amalgam restorations also showed less marginal breakdown, potentially leading to a longer-lasting restoration. Additionally, the bonded amalgam restorations demonstrated higher retention, reduced microleakage, and less post-operative sensitivity compared to conventional amalgam restorations. Based on these findings, the researchers recommended that all future amalgam restorations be bonded to enhance their retentive strength and increase their longevity [17-19].

These findings align with the results reported by Opdam et al., despite using different materials for testing. When bonding amalgam to dentin, glass ionomer cements can serve as an effective bonding agent due to their chemical adhesion to the tooth surface. If the glass ionomer material is used in bonded amalgam restorations before it has fully set, the unset amalgam projections enhance micromechanical retention by creating tag formations that ultimately strengthen the bond. This improved bond leads to better retentive values for the amalgam restorations. One of the primary objectives of an ideal restoration is to be securely retained within the cavity with sufficient strength to withstand occlusal forces. This goal can be achieved through the use of bonded amalgam restorations [18,19].

The retentive strength of amalgam-bonded restorations relies on the bonding capacity of the bonding material to both the tooth and the amalgam restoration itself. In a five-year study on complex amalgam restorations, the bonding of amalgam proved to be a feasible alternative compared to the mechanical retention achieved with traditional restorations [20].

When bonding amalgam restorations to dentin, the amalgam bonding agent or a similar product can serve as an efficient sealing agent, reducing issues like microleakage, dentin hypersensitivity, and secondary decay at the interface between the tooth and the restoration. Additionally, using an amalgam bond or another bonding agent with amalgam restorations offers the advantage of increased bond strength resulting from the procedure.

Limitations

The limitations of the study include factors such as the limited time frame of the follow-up, potential bias in patient selection, a small sample size, and the absence of long-term observations that might be necessary to assess the durability and stability of the bonded amalgam restorations over extended periods. Additionally, the study might not account for confounding variables that could influence the results, such as oral hygiene habits, dietary factors, or other existing dental conditions. Therefore, it is recommended that future studies on the same theme take these limitations into account.

## Conclusions

Based on the study's findings, it can be concluded that bonded amalgam restorations exhibit superior retention and better marginal strength compared to conventional amalgam restorations. Various materials can be utilized for bonding amalgam restorations, some of which show significant advantages in bonding amalgam restorations due to their high retentive strength, reduced microleakage, and potential to minimize secondary caries risk. Therefore, it is recommended that all amalgam restorations be bonded to enhance retention, reduce microleakage, and prolong their lifespan in the oral environment.
